# Development and External Validation of a Prediction Model to Identify Suicide Attempters in Treatment‐Naive Adolescents With Major Depressive Disorder

**DOI:** 10.1155/da/7216497

**Published:** 2026-02-27

**Authors:** Yuqin Song, Mengqin Dai, Qiuyue Fan, Lu Pan, Yuhang Wu, Jiarui Shao, Cen Lin, Wenxiu Luo, Yu Cen, Cailin Xie, Xiangli Wang, Jiaming Luo

**Affiliations:** ^1^ Mental Health Center, Affiliated Hospital of North Sichuan Medical College, Nanchong, Sichuan, China, hospital-nsmc.com.cn; ^2^ School of Psychiatry, North Sichuan Medical College, Nanchong, Sichuan, China, nsmc.edu.cn; ^3^ Center for Vocational Education Research, Shenzhen University of Information Technology, Shenzhen, Guangdong, China; ^4^ Key Laboratory of Digital-Intelligent Disease Surveillance and Health Governance, North Sichuan Medical College, Nanchong, Sichuan, China, nsmc.edu.cn

**Keywords:** adolescents, depression, machine learning, prediction model, suicide attempt

## Abstract

**Background:**

Suicidal behavior in adolescents poses a significant risk, and suicide attempts are the strongest predictors of suicide death. Patients with Major Depressive Disorder (MDD) are at high risk of attempting suicide. However, there is still a lack of effective tools in clinical settings to identify these suicide attempters.

**Methods:**

The study assessed suicidal attempts and their predictive factors in adolescents first diagnosed with MDD from August 1, 2022, to May 31, 2024. Five algorithms were used for model construction: logistic regression, random forest, decision tree, support vector machine, and XGBoost. Finally, we evaluated the performance of the best model using an independent external validation set.

**Results:**

The study included 820 untreated adolescent first‐visit MDD patients (618 females [75.4%], average age 14.67 ± 1.69 years). Of these, 481 (58.7%) had disclosed suicidal ideation to others, and 299 (36.5%) reported having attempted suicide. Predictive variables for the outcome included age, grade, BMI z‐score, levels of depression and anxiety, sleep quality, history of being left behind, father’s occupation, primary residence before age 16, history of non‐suicidal self‐injury (NSSI) within the last year, and history of disclosure of suicidal ideation. The XGBoost model showed the highest prediction accuracy (ROC_AUC, 0.72; PR_AUC, 0.65) and sensitivity (0.85) after external validation. The history of NSSI within the last year had the strongest predictive effect on suicide attempts, followed by disclosure of suicidal ideation, sleep quality, BMI z‐score, and anxiety levels.

**Conclusions:**

Despite including only 11 easily collectible clinical variables, the XGBoost model effectively identifies suicide attempters among untreated adolescent first‐visit MDD patients and performs stably in external validation sets. This is beneficial for clinicians to conduct evidence‐based suicide prevention efforts.

## 1. Introduction

Suicide is a significant public health issue and the second leading cause of death among children and adolescents aged 10–24 [1]. In China, ~10,000 adolescents die by suicide each year, accounting for 19% of all adolescent deaths [2]. Research has indicated that the incidence of suicide increases rapidly from late childhood to early adolescence, peaking in mid‐to‐late adolescence, and then stabilizes or declines in early adulthood [3]. Consequently, adolescence is a crucial period for studying suicidal behaviors. Patients with depressive disorders are particularly prone to suicide [4] and related behaviors [5–7], and in recent years, the onset age of depressive disorders has been trending younger [8]. The “China National Mental Health Development Report” found that the detection rate of depressive symptoms among Chinese adolescents was 24.6% in 2020 [9]. Therefore, research on suicidal behaviors among adolescents with depressive disorders is urgently needed.

Deaths from suicide typically occur after multiple suicide attempts [10], and suicide attempts are considered the most powerful predictors of suicide death [11–13]. However, previous research on suicide has often focused on suicidal ideation, with many studies finding that variables like depression and despair are highly correlated with suicidal ideation but not as effective in predicting suicide attempts [14]. The predictive role of suicidal ideation for suicide attempts remains unclear, with the World Health Organization finding that about two‐thirds of those with suicidal ideation never attempt suicide [15]. Research by Moro et al. [16] suggests that only 7.8% of those with suicidal ideation will attempt suicide within 2 years. Beyond suicidal ideation, several other factors have been associated with suicide attempts. For instance, studies have demonstrated that sleep disturbances are closely related to suicide attempts, and this association remains significant even after controlling for depressive symptoms [17]. Body mass index (BMI) has also been linked to suicidal behavior; although findings are inconsistent, some studies suggest that both low and high BMI may increase the likelihood of suicide attempts [18]. Anxiety symptoms, particularly at higher levels, may contribute to suicide attempts by intensifying psychological distress and impulsivity [19]. Notably, these variables are readily obtainable in clinical practice, highlighting their potential utility. Developing prediction models based on such easily accessible indicators may help clinicians identify individuals at high risk for suicide attempts early, without the need for complex assessments.

In recent years, machine learning algorithms have been used to analyze suicidal behavior, especially through large‐scale data such as electronic medical records [20]. Compared with traditional methods, machine learning algorithms can automatically capture the nonlinear relationships between variables and outcome events, whereas traditional algorithms may lead to misjudgments due to insignificant linear correlations, thus missing some important variables [21]. Meanwhile, machine learning excels at handling high‐dimensional data, a feature that helps us better process complex real‐world clinical data [22]. In addition, the value of constructing machine learning models lies not only in revealing the associations between variables and outcomes but also in their potential to be developed into complete and usable clinical decision support tools. When combined with SHAP analysis, they can visualize how individual variables influence outcomes [23].

Previous studies on suicide attempts have been limited, often based on the general population, with fewer studies focused on high‐risk groups like those with MDD, and involved many variables, which increases patient burden and diminishes clinical utility. Many models lack external validation, making it difficult to assess their applicability and stability in different clinical settings, thereby reducing their credibility and reliability for widespread use. Therefore, this study used data from two centers and compared five algorithms with the aim of using easily obtainable clinical information to construct a risk prediction model for suicide attempts among adolescent MDD patients and to externally validate it, thereby reducing patient burden at the point of care and facilitating early and precise suicide prevention measures. This study also explores important predictive factors for suicide attempts in this population.

## 2. Methods

### 2.1. Participants

**Figure 1:**
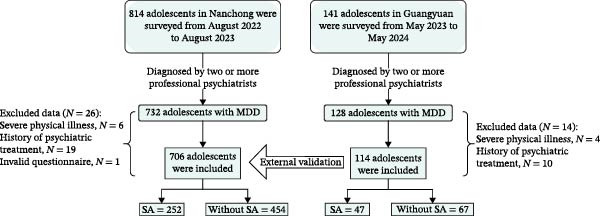
Data source.

Before the study commenced, standardized operating procedures were established, and uniform training was provided to the graduate students and physicians participating in the research. Using a continuous enrollment approach, 814 adolescent patients (aged 10–18) first diagnosed with Major Depressive Disorder (MDD) at the outpatient department of the Affiliated Hospital of North Sichuan Medical College in Nanchong, Sichuan, from August 2022 to August 2023, were included. Researchers explained the purpose, content, and confidentiality of the survey to the subjects and their parents and obtained informed consent. Each subject was diagnosed with MDD by two independent professional psychiatrists according to the Diagnostic and Statistical Manual of Mental Disorders, Fifth Edition (DSM‐5) criteria, using the Mini International Neuropsychiatric Interview for Children and Adolescents (MINI‐KID). The diagnoses were made through consensus between the two psychiatrists. All self‐report measures were administered by trained graduate students under the supervision of licensed clinical psychologists in a quiet, private room within 24 h of the diagnostic interview, and participants were given sufficient time to complete the questionnaires independently. The inclusion and exclusion criteria are detailed in Appendix S1 of the Supporting Information. Using the same method, from May 2023 to May 2024, 141 adolescent patients (aged 10–18) first diagnosed with MDD at the outpatient department of the Mental Health Center in Guangyuan City were included. After dual screening, 114 subjects were finally included as external validation data for the model (Figure 1). This study was approved by the Medical Ethics Committee of the Affiliated Hospital of North Sichuan Medical College and was registered with the Chinese Clinical Trial Registry (registration number ChiCTR2200064623). This study follows the Transparent Reporting of a multivariable prediction model for Individual Prognosis Or Diagnosis (TRIPOD) reporting guidelines for the development of prediction models [24].

### 2.2. Measures

#### 2.2.1. Assessment of Suicide Attempts

In this study, we used specific items from the Suicidal Behaviors Questionnaire‐Revised (SBQ‐R) to document historical suicidal events rather than to conduct comprehensive suicide risk screening. The SBQ‐R was developed by [25] as a brief assessment tool for suicidal behaviors [25]. Importantly, individual items from the SBQ‐R have been validated for retrospective assessment of specific suicidal events in previous research [26]. Item 1 (“Have you ever considered or attempted suicide?”) was used to assess lifetime history of suicide attempts. Responses of “I have attempted suicide but did not really want to die” and “I have attempted suicide and really wanted to die” were both classified as having a history of suicide attempts, consistent with established definitions of suicide attempts that include acts regardless of lethality or intent to die. The use of individual SBQ‐R items for documenting specific historical events has been supported by previous studies demonstrating that these items maintain adequate test–retest reliability (kappa > 0.70) and convergent validity with clinician‐rated assessments when used independently. This approach aligns with recommendations that single‐item measures can be appropriate for assessing well‐defined, unambiguous constructs such as lifetime history of specific behaviors.

#### 2.2.2. Assessment of Predictors

Based on a review of the existing literature, we identified a set of potential predictive variables and, guided by principles of clinical feasibility, ultimately selected 21 variables for evaluation [5, 14, 27, 28]. These variables, routinely collected in clinical settings, included age, gender, whether the medical visit was voluntary, whether there was an experience of being left behind, parents’ marital status, whether the individual was a boarding student, whether they were an only child, grade level, parents’ educational level, parents’ occupations, primary residence before age 16, family history of mental illness, and academic performance ranked within their class. The calculation of Body Mass Index Z‐scores (BMIZs) was performed using the WHO Anthro Plus software [29, 30]. Depressive symptoms were evaluated using the Chinese version of the Self‐Rating Depression Scale (SDS) [31, 32], with a Cronbach’s alpha of 0.796 in the current sample. Anxiety symptoms were assessed using the Chinese version of the Self‐Rating Anxiety Scale (SAS) [33, 34], yielding a Cronbach’s alpha of 0.881. Sleep quality was measured using the Chinese version of the Pittsburgh Sleep Quality Index (PSQI) [35, 36], with a Cronbach’s alpha of 0.855 in this study. Non‐suicidal self‐injury (NSSI) was assessed based on the DSM‐5 definition with the item, “In the past year, have you engaged in any intentional, direct harm to your body without suicidal intent?” [37, 38]. A “yes” response was considered as having a history of NSSI. Item 3 of the SBQ‐R (“Have you ever told someone about your intention to commit suicide or thoughts of possibly committing suicide?”) was used to assess lifetime disclosure of suicidal ideation, with any response other than “never” indicating the presence of disclosure of suicidal ideation.

### 2.3. Statistical Analysis

Data analysis was conducted using R software (version 4.4.1). Normality tests for numerical variables were performed using the Shapiro–Wilk test in combination with Q–Q plots. Normally distributed variables were expressed as mean ± standard deviation and compared between groups using independent two‐sample *t*‐tests. Skewed numerical variables were represented by the median (interquartile range) and compared between groups using the Mann–Whitney *U* test. Categorical variables were expressed as n% (percentage) and compared using the chi‐square test. In this study, the missing rate for all variables did not exceed 5%. Missing data were imputed using the Random Forest algorithm, which does not require any assumptions regarding the underlying data distribution. All categorical variables were one‐hot encoded, and multicollinearity among predictors was assessed using the variance inflation factor (VIF), confirming the absence of serious multicollinearity.

All 706 samples from the Nanchong center were used for model development with the presence of a suicide attempt as the outcome variable and the other 21 variables as candidate predictors. To prevent data leakage and ensure unbiased performance estimation, all modeling procedures—including feature selection, hyperparameter tuning, and model training—were performed within a nested cross‐validation framework.

Specifically, the dataset was divided into 5 folds for outer cross‐validation. For each fold, the remaining 4 folds (~80% of the data) served as the training set, while the held‐out fold (~20%) served as the validation set. Within each training set, feature selection was independently conducted using two complementary approaches: (1) the Boruta algorithm based on random forests to identify confirmed and tentative important variables (see Figure S1A in the Supplement), and (2) XGBoost feature importance ranking to select the top 10 contributing variables (see Figure S1B in the Supplement). The union of these two sets resulted in 11 predictor variables for each fold (for detailed operational definitions and categorization of variables, please refer to Table S1 in the Supporting Information). Importantly, feature selection was performed de novo within each outer fold using only the training data from that fold, ensuring that the validation set remained completely unseen during this process.

Subsequently, within each training fold, hyperparameter optimization was conducted using an inner 5‐fold cross‐validation with grid search. Five machine learning algorithms were employed: logistic regression, random forest, decision tree, support vector machine, and XGBoost (optimal hyperparameters are detailed in Supporting Information Table S3). After identifying the optimal hyperparameters, the final model for each fold was trained on the complete training set of that fold and evaluated on the corresponding held‐out validation set. This nested cross‐validation procedure was repeated across all 5 outer folds, and performance metrics were averaged to obtain unbiased estimates of model performance.

For external validation, the final prediction model was constructed using the entire 706‐sample training dataset with the same feature selection strategy applied to the full dataset, and the model was then evaluated on the independent external validation cohort of 114 patients from Guangyuan City. A 1000‐times bootstrap resampling was performed on both the training set and the external validation set to evaluate the model’s performance.

Considering the class imbalance in the outcome events, model performance was evaluated using the ROC (Receiver Operating Characteristic) curve and the PR (Precision‐Recall) curve. Additionally, sensitivity, specificity, positive predictive value (PPV), negative predictive value (NPV), and F1 score of the best‐performing model on the external validation set were reported (see Table S2 in the Supporting Information for definitions of these metrics). Calibration analysis was visualized using a locally estimated scatterplot smoothing (LOESS) method, and DCA was used to evaluate the clinical utility of the model. The default feature importance in machine learning models often assigns higher contribution scores to continuous or high‐cardinality [39] categorical variables. To address this, we employed permutation‐based importance [40, 41] to provide a global interpretation of the model, emphasizing the overall contribution of each feature. Additionally, we conducted SHAP (Shapley Additive Explanations) analysis for a local interpretation, assessing the impact of each feature on individual predictions.

## 3. Results

### 3.1. Prevalence of Suicide Attempts

This study included a total of 820 adolescent patients aged 10–18 years, with an average age of 14.67 ± 1.69 years, of whom 618 (75.4%) were female. Of these, 299 (36.5%) reported having attempted suicide, and 481 (58.7%) had disclosed suicidal thoughts to someone in the past year. Additionally, 553 (67.4%) reported engaging in NSSI. Except for differences in BMI z‐score, father’s occupation, and primary residence before the age of 16, there were no significant differences in other characteristics between the external validation set and the development set. Sociodemographic characteristics of the sample are presented in Table S4.

### 3.2. Model Performance

**Figure 2:**
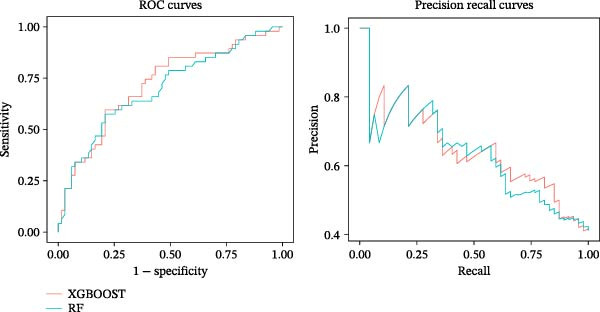
Performance of XGBoost and random forest models on the external validation set. XGBoost achieved a ROC_AUC of 0.72 (95% CI: 0.57–0.85) and a PR_AUC of 0.65 (95% CI: 0.44–0.82), while Random Forest achieved a ROC_AUC of 0.70 (95% CI: 0.55–0.83) and a PR_AUC of 0.64 (95% CI: 0.43–0.81). The results were validated through 1000 bootstrap resamples.

On the training set, the Random Forest model showed the best performance with a ROC_AUC of 0.82 (95% CI: 0.77–0.86) and a PR_AUC of 0.69 (95% CI: 0.61–0.77), while the XGBoost model followed closely with a ROC_AUC of 0.79 (95% CI: 0.75–0.84) and a PR_AUC of 0.65 (95% CI: 0.57–0.74) (Table 1). On the external validation set, the XGBoost model performed the best, with a ROC‐AUC of 0.72 (95% CI: 0.57–0.85) and a PR‐AUC of 0.65 (95% CI: 0.44–0.82), and the Random Forest model yielded a slightly lower ROC_AUC of 0.70 (95% CI: 0.55–0.83) and PR_AUC of 0.64 (95% CI: 0.43–0.81) (Figure 2). To verify whether the performance differences between the two models were statistically meaningful, we conducted formal statistical comparisons: DeLong test for ROC_AUC (*p* = 0.168) and Bootstrap test (1000 resamples) for PR_AUC (*p* = 0.152). The results indicated that the differences in ROC_AUC and PR_AUC between the two models did not reach statistical significance. Regarding sensitivity (a key indicator for minimizing false negatives in clinical screening tools), the XGBoost model exhibited a significantly higher sensitivity of 0.85 (95% CI: 0.71–1.00), compared to 0.65 (95% CI: 0.51–0.80) for the Random Forest model. A proportion test confirmed that this sensitivity difference was statistically significant (*p* = 0.028).

**Table 1:** The model’s performance on the training set.

Models	ROC_AUC (95%CI)	PR_AUC (95%CI)
Logistic regression	0.74 (0.69–0.79)	0.58 (0.49–0.67)
Decision tree	0.73 (0.68–0.78)	0.56 (0.47–0.64)
Random torest	0.82 (0.77–0.86)	0.69 (0.61–0.77)
XGBoost^a^	0.79 (0.75–0.84)	0.65 (0.57–0.74)
SVM^b^	0.68 (0.63–0.74)	0.53 (0.45–0.62)

*Note:* The results were validated through 1000 bootstrap resamples.

^a^Extreme gradient boosting.

^b^Support vector machine.

Although the overall discriminative performance (ROC_AUC and PR_AUC) of the two models was not significantly different on the external validation set, the XGBoost model was ultimately selected as the final model. This decision was primarily based on its significantly superior sensitivity, which is crucial for a clinical screening tool aiming to maximize the identification of target cases and reduce the risk of missed diagnosis in clinical practice.

Given the class imbalance in the data of this study, we also comprehensively reported other performance metrics of the XGBoost model on the external validation set: specificity 0.48 (95% CI: 0.31–0.63), PPV 0.53 (95% CI: 0.38–0.68), NPV 0.82 (95% CI: 0.64–1.00), and F1 score 0.65 (95% CI: 0.51–0.70). The calibration curve indicates that the predicted probabilities of the XGBoost model align well with the observed positive rates (Figure S2), with an Integrated Calibration Index (ICI) of 0.0576, suggesting minimal calibration bias. The clinical DCA indicates that the model has good application value in the low‐to‐moderate threshold range (Figure S3).

### 3.3. Model Explanation

**Figure 3:**
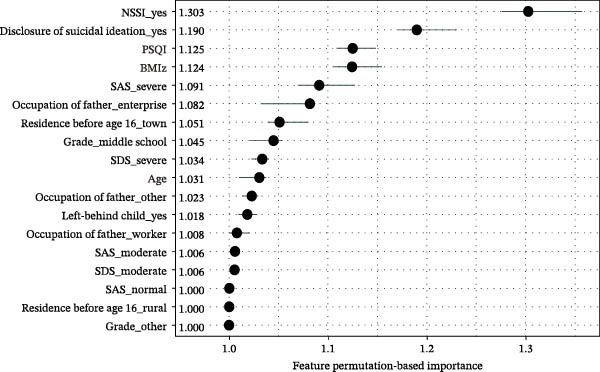
The permutation‐based importance plot. SAS evaluates anxiety, SDS evaluates depression, and PSQI evaluates sleep quality.

**Figure 4:**
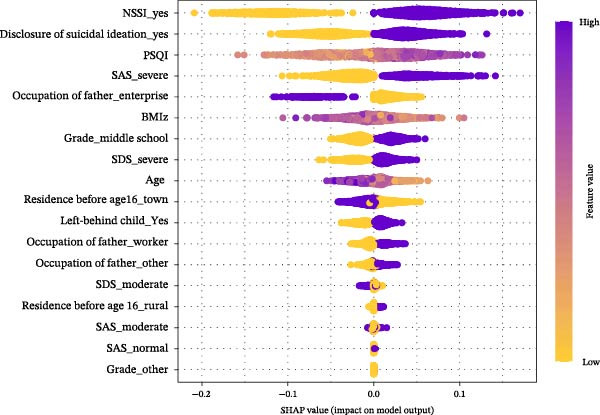
The SHAP summary plot. SAS evaluates anxiety, SDS evaluates depression, and PSQI evaluates sleep quality. The vertical axis ranks features by importance (top to bottom). Each point’s position on the horizontal axis shows its impact on a prediction (right: positive; left: negative), while color represents the feature value (purple: high; yellow: low).

The permutation‐based importance plot (Figure 3) shows that the top 5 important variables are the history of NSSI, disclosure of suicidal ideation, sleep quality, BMI z‐score, and anxiety. The SHAP summary plot (Figure 4) illustrates the specific impact of each feature. Individuals with a history of NSSI, disclosure of suicidal ideation, poor sleep quality, severe anxiety, lower BMI z‐score, and younger age are more likely to be predicted as suicide attempters.

## 4. Discussion

In this study, we developed a clinically applicable machine learning‐based model to predict suicide attempts in treatment‐naive adolescents with MDD and validated its robustness using external datasets. To our knowledge, this represents one of the first predictive models specifically targeting untreated adolescents with MDD. Our findings further identified that a history of NSSI, disclosure of suicidal ideation, sleep quality, BMI z‐score, and anxiety symptoms are key predictors of suicide attempts in this population. These results not only advance the understanding of mechanisms underlying suicide attempts among adolescents but also provide a theoretical basis for the development of individualized intervention strategies.

This study demonstrated that the XGBoost model could achieve high sensitivity and robust generalizability even when using a limited number of predictors. This advantage is primarily attributable to the model’s gradient boosting framework and regularization design, which optimize performance iteratively while controlling model complexity. A large body of evidence has also confirmed that XGBoost consistently exhibits superior performance across various clinical prediction models [42]. Previous studies often relied on numerous variables and multiple scales to enhance predictive performance [43–45], which considerably reduced clinical feasibility. For example, a 2023 nationwide study incorporated 169 questionnaire items to construct an XGBoost prediction model [46]. Meanwhile, a growing body of evidence suggests that models with fewer variables can also perform well. A recent meta‐analysis [47] indicated that models developed using a smaller set of variables can still achieve excellent predictive performance (ROC_AUC > 0.70), although most lacked external validation. Reducing the number of predictors not only enhances the model’s generalizability and interpretability but also simplifies real‐world data collection, thereby improving clinical applicability. In practice, adolescents presenting for an initial visit or diagnosed with MDD can complete an 11‐item electronic questionnaire on a hospital terminal within approximately 5 minutes, allowing rapid identification of high‐risk individuals without adding burden to clinicians. The model outputs not only a risk score but also individualized risk profiles generated through SHAP analysis, guiding clinicians to focus on relevant areas during interviews and enhancing interview efficiency. The final risk assessment should integrate model outputs with standard clinical evaluation to inform personalized intervention strategies.

This study also found that among the adolescent MDD population, the incidence of suicide attempts reached as high as 36.5%. Some studies have shown that patients with severe depressive disorder (MDD) have a lifetime prevalence of suicide attempts around 31% [48], and a TORDIA study reported a lifetime suicide attempt rate of 23.5% in adolescents with treatment‐resistant depression [49]. In previous studies, the rate of suicide attempts among the general adolescent population was found to be between 7 and 9% [50, 51]. This indicates that adolescents with depressive disorders are more prone to attempting suicide. However, research specifically addressing suicide attempts among adolescents with depressive disorders is limited. The variation in rates of suicide attempts among adolescents with depressive disorders in different studies can be attributed mainly to two factors. First, different studies define suicide attempts and related behaviors differently. It is known that self‐harm and suicide attempts are strongly linked, but many studies do not distinguish between the two [27]. Second, the stigma associated with suicide can affect reporting results, and religious and cultural differences in different regions can also impact the accuracy of reports [52].

In this study, NSSI emerged as the strongest predictor of suicide attempts, consistent with previous research. Studies indicate that among individuals who engage in NSSI, 6% to 37% subsequently attempt suicide, and among those who attempt suicide, 41% to 68% have a history of NSSI [53]. A large body of evidence has confirmed a significant association between NSSI and suicide attempts, with the risk of suicide attempts increasing alongside the frequency or severity of NSSI episodes [13]. Furthermore, multiple longitudinal cohort studies have found that the occurrence of NSSI often precedes suicide attempts [53]. Joiner et al. [54] proposed that repeated engagement in NSSI, especially with increasing severity, gradually desensitizes individuals to pain, injury, and the fear of death. This notion is further supported by Klonsky and May [55], who suggested that both pain tolerance and fearlessness about death constitute components of the acquired capability for suicide, which is a key factor in the transition from suicidal ideation to suicide attempts. Taken together, NSSI likely serves as a critical predictor of suicide attempts by facilitating the development of acquired capability for suicide.

Our findings indicate that disclosure of suicidal ideation is the second strongest predictor of suicide attempts. Influenced by cultural and social contexts, only 30%–50% of individuals experiencing suicidal ideation actually disclose it to others [56]. Unlike suicidal ideation, which is an internal psychological process, disclosure represents an externalized help‐seeking signal, the effectiveness of which depends on the response and support provided by the recipient. Studies have shown that family members are the most common recipients of such disclosures, particularly for young people, as family not only serves as a critical source of emotional support but can also facilitate access to professional help, such as medical appointments or referrals [57]. Conversely, if adequate support is not provided following disclosure, individuals may experience exacerbated psychological distress and hopelessness [58]. Therefore, disclosure of suicidal ideation serves not only as an important signal for suicide intervention but also as a crucial step in the transition from ideation to action. Future research should explore how young people choose whom to disclose their suicidal thoughts to and the effects of such disclosure on both parties. Further investigation into optimizing family and social support systems could inform targeted clinical interventions.

Our findings further confirm that poor sleep quality significantly increases the risk of suicide attempts. Sleep quality encompasses multiple dimensions, yet only insomnia and nightmares have been found to be significantly associated with elevated risk of suicidal behavior [59]. Among individuals with depressive disorders, severe insomnia has been identified as one of the few clinical predictors of suicidal behavior within the first year of follow‐up [60]. Fang et al. [61] reported that delayed insomnia was closely associated with suicidal ideation in Chinese patients with first‐episode MDD. Other studies have found that nightmares are more prevalent among patients with depressive disorders who have previously attempted suicide [62]. Dysfunction of the serotonergic system (5‐hydroxytryptamine, 5‐HT) is one of the key mechanisms linking sleep disturbances to suicidal behavior in individuals with depression. The main metabolite of 5‐HT, 5‐hydroxyindoleacetic acid (5‐HIAA), has been shown to be significantly reduced in the cerebrospinal fluid of depressed patients, correlating with an increased risk of suicidal behavior [63]. Additionally, it has been suggested that sleep may provide patients with depression a temporary alternative to suicide, allowing them to escape daily life stressors. However, various sleep disturbances may prevent patients from achieving relief through sleep, thereby increasing the likelihood of attempting suicide as a means to alleviate psychological distress [64].

In this study, depressive symptoms ranked relatively low in importance for predicting suicide attempts, placing only eighth among all predictors. This phenomenon may be explained by several factors. First, although the severity of depression is a key predictor of suicide attempts in the general population [65], adolescents with MDD typically present with uniformly high depression scores at baseline (91.5% of participants exhibited moderate‐to‐severe depression), reducing the discriminatory power of the measure. Second, individuals with severe depression may lack the motivation or energy required to enact suicidal behavior, thereby lowering the immediate risk of suicide attempts [54]. Finally, depressive symptoms in this study were assessed using self‐report scales; clinician‐administered measures, such as the Hamilton Depression Rating Scale or the Montgomery–Åsberg Depression Rating Scale, may provide more reliable assessments.

This study also found that anxiety symptoms exhibited substantial predictive value for suicide attempts in adolescents with MDD, consistent with previous research. Although depression and anxiety frequently co‐occur, anxiety retains independent predictive value for suicide attempts even after controlling for depressive severity [66]. A longitudinal study further demonstrated that, after adjusting for other psychiatric disorders, the presence of an anxiety disorder increased the risk of suicidal behavior by up to 2.8‐fold [67]. Individuals with severe anxiety are more likely to engage in negative cognitive patterns and hold pessimistic views of the future, thereby significantly elevating the risk of suicide attempts [68]. Accordingly, clinical interventions should pay particular attention to patients’ anxiety symptoms and incorporate targeted strategies. For instance, alleviating anxiety through cognitive–behavioral therapy (CBT) or pharmacological treatment may more effectively reduce the risk of suicide.

## 5. Limitations

Although this study employed external validation, the moderate level of discrimination and the relatively homogeneous validation cohort still limit the generalizability of the model, leaving a potential risk of overfitting. Additionally, all suicide‐related variables in this study were based on self‐report, which may be subject to recall bias and social desirability bias. We also observed that some predictors exhibited nonlinear relationships with suicide attempts, suggesting that increasing the sample size may help to reveal more stable and clearer association patterns. Furthermore, as this study employed a cross‐sectional design, treating suicide attempts as the outcome variable is somewhat controversial; if suicide attempts are considered events occurring at a specific time point [69], it may not be appropriate to predict them using a diagnostic prediction model. However, given that past suicide attempts are strong predictors of future suicide attempts [70, 71], conceptualizing them as an individual trait—that is, classifying individuals with a history of suicide attempts as positive cases for predictive purposes—is reasonable [27]. Future research should adopt prospective cohort designs to examine the occurrence and prediction of suicide attempts over shorter timeframes, for example, using ecological momentary assessment (EMA) methods [72], which can both evaluate the effectiveness of interventions and elucidate the dynamic progression from suicidal ideation to suicide attempts. Moreover, the model’s predictive performance for positive cases still has room for improvement. Our results indicated relatively small performance differences among different machine learning models, particularly with large sample sizes. Future studies could further explore features and clinical symptoms associated with high‐value predictors and integrate multimodal data, such as biological markers and neuroimaging, to enhance the objectivity and predictive performance of the model. Adolescents with depressive disorders are inherently at high risk for conversion to bipolar disorder; therefore, future research should incorporate standardized screening tools, such as the Mood Disorder Questionnaire (MDQ) or the Hypomania Checklist‐32 (HCL‐32), to improve identification of potential bipolar trajectories. From a scientific perspective, observing and assessing the natural course of suicide attempts is crucial, but once a participant reports a suicide attempt, researchers have an ethical obligation to intervene. In this study, crisis hotlines and professional crisis intervention resources were provided to individuals with suicide attempts, which, although affecting natural observation, was ethically indispensable. Finally, the predictive model developed in this study could be implemented as a mobile application (App), enhancing the practical utility of the research findings and allowing for the incorporation of new data in future studies to further validate and optimize the model.

## 6. Conclusions

The diagnostic prediction model developed in this study using machine learning demonstrated that incorporating a limited number of predictors can effectively assist in identifying adolescents at risk of having a history of suicide attempts. The analysis revealed that a history of NSSI, disclosure of suicidal ideation, sleep quality, BMI z‐score, and anxiety symptoms were the most influential predictors in the current model. Future research should consider integrating biochemical and neuroimaging markers to further identify more objective and reliable predictors with greater predictive potential, thereby optimizing risk assessment tools. Moreover, given the retrospective design of this study, prospective studies are still required to validate the model before it can be applied in clinical practice.

## Funding

This research was funded by Scientific Research Project of Nanchong Federation of Social Science Associations (Grant NC24A016) and Scientific Research Project of Primary Health Care Research Center of Sichuan Province (Grant SWFZ24‐Z‐04).

## Disclosure

The content is solely the responsibility of the authors.

## Conflicts of Interest

The authors declare no conflicts of interest.

## Supporting Information

Additional supporting information can be found online in the Supporting Information section.

## Supporting information


**Supporting Information** To further support the completeness of the article, we provide the following materials in the supporting section: Appendix S1: Inclusion and exclusion criteria for participants. Figure S1: Feature selection process using Boruta and XGBoost methods. Table S1: Definitions and classification categories of study variables. Table S2: Glossary of Statistical and Machine Learning Model Evaluation Metrics & Interpretation Methods. Table S3: Overview of all machine learning algorithms used in the study, along with their optimal parameters. Table S4: Description of participants’ clinical characteristics. Table S5: All performance evaluation metrics of the XGBoost model on the external validation set. Figure S2: Calibration curve of the model. Figure S3: Clinical decision curve analysis of the model.

## Data Availability

The data that support the findings of this study are available upon request from the corresponding author. The data are not publicly available due to privacy or ethical restrictions.
