# Supplementary material for: Development and External Validation of a Prediction Model to Identify Suicide Attempters in Treatment‐Naive Adolescents With Major Depressive Disorder
Source: Depress Anxiety. 2026 Feb 27;2026:7216497. doi: 10.1155/da/7216497 (PMC12947665; doi:10.1155/da/7216497)
Supplement: Supplementary file 1 — Supporting Information To further support the completeness of the article, we provide the following materials in the supporting section: Appendix S1: Inclusion and exclusion criteria for participants. Figure S1: Feature selection process using Boruta and XGBoost methods. Table S1: Definitions and classification categories of study variables. Table S2: Glossary of Statistical and Machine Learning Model Evaluation Metrics & Interpretation Methods. Table S3: Overview of all machine learning algorithms used in the study, along with their optimal parameters. Table S4: Description of participants’ clinical characteristics. Table S5: All performance evaluation metrics of the XGBoost model on the external validation set. Figure S2: Calibration curve of the model. Figure S3: Clinical decision curve analysis of the model. [file DA-2026-7216497-s001.docx]

**Appendix S1:**

**Inclusion Criteria:**

1. Age between 10-18 years.
2. First-episode MDD diagnosis confirmed by DSM-5 criteria via MINI-KID.
3. Ability to understand and complete questionnaires.
4. Written informed consent from both participants and their legal guardians.

**Exclusion Criteria:**

1. Participants with significant cognitive impairment or functional limitations prevent comprehension of the content of questionnaire.
2. Presence of severe physical diseases or other neurological disorders.
3. Comorbid bipolar disorder, schizophrenia, or other severe mental disorders.

**Figure S1: Feature Selection**

**
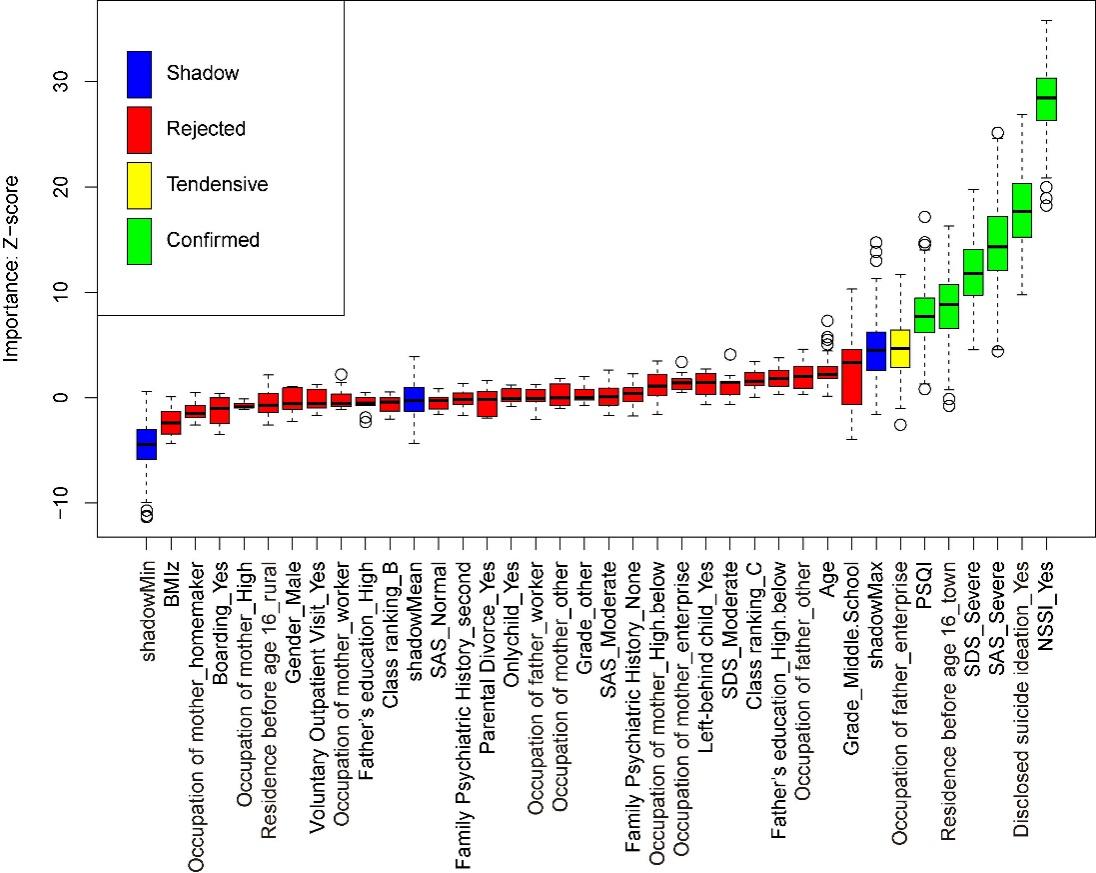
A: Boruta-Based Feature Selection**

**
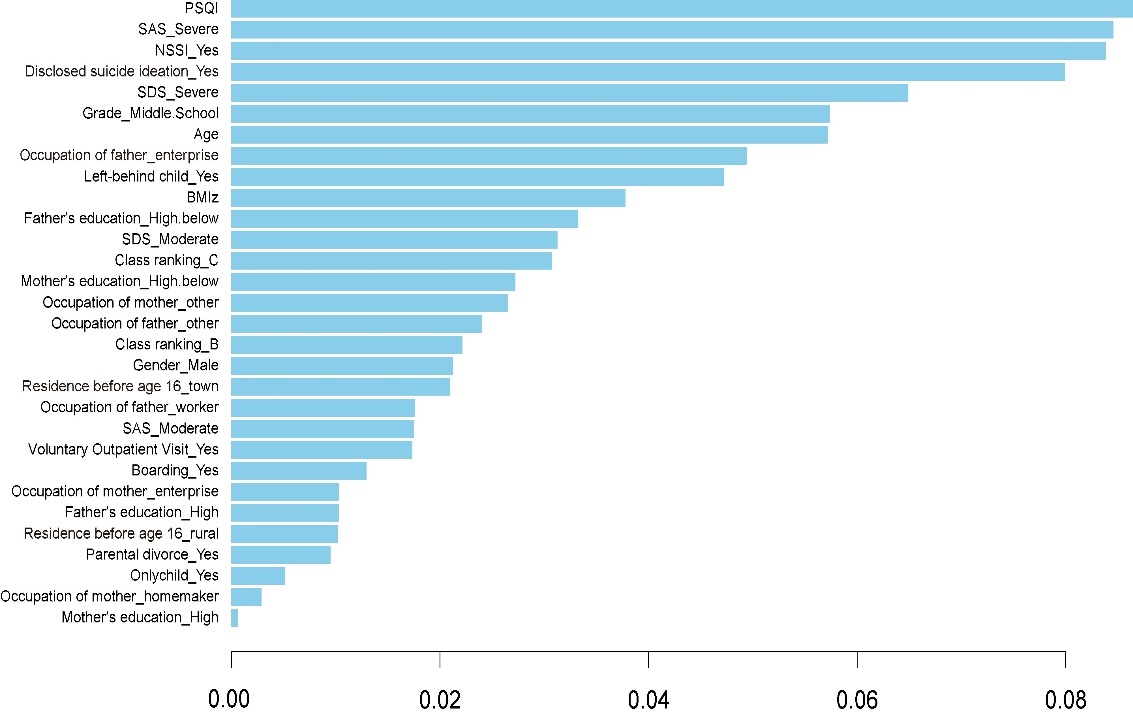
B: XGBoost Feature Importance for All Variables**

Variables selected in Figure A include: non-suicidal self-injury, disclosed suicide ideation, anxiety, depression, primary residence before age 16, Pittsburgh Sleep Quality Index, occupation of father.

The top 10 most important variables in Figure B include: Pittsburgh Sleep Quality Index, anxiety, non-suicidal self-injury, disclosed suicide ideation, depression, grade, age, occupation of father, left-behind child, BMI z-score.

**Table S1:** **Definitions and classification categories of study variables**

| **Variable Name** | **Variable Type** | **Categories/Values and Interpretation** |
| --- | --- | --- |
| Age | Continuous | Actual chronological age of participants, unit: years |
| Grade | Categorical | Middle School; High School; other |
| BMIz | Continuous | Body Mass Index z-score, standardized by age and gender to reflect deviation from population mean |
| Occupation of Father | Categorical | Worker; Business; Enterprise; Other; |
| Residence(Primary Residence before Age 16) | Categorical | Rural; Town; City |
| SDS (Depression Levels) | Categorical | Severe; Moderate; Mild  (Its severity is graded according to the standard score, where 53-62 points is mild, 63-72 points is moderate, and 73 points and above is severe.) |
| SAS (Anxiety Levels) | Categorical | Severe; Moderate; Mild; Normal  (Its severity is graded according to the standard score, with scores below 50 considered normal, 50-59 points indicating mild anxiety, 60-69 points indicating moderate anxiety, and 70 points and above indicating severe anxiety.) |
| PSQI (Pittsburgh Sleep Quality Index) | Continuous | Composite score of sleep quality; higher scores indicate more severe sleep problems |
| LBC (Experience of Being Left Behind) | Categorical | Yes (experience of being separated from both parents or either parent continuously for at least 6 months within one year due to parental migration for work);  No (no such left-behind experience) |
| NSSI (Non-Suicidal Self-Injury) | Categorical | Yes; No |
| Disclosure of Suicidal Thoughts | Categorical | Yes; No |
| SA (Suicide Attempt) | Categorical | Yes ; No |

All categorical variables were encoded using one-hot encoding.

**Table S2:** **Glossary of Statistical and Machine Learning Model Evaluation Metrics & Interpretation Methods**

| **Term** | **Explanation** |
| --- | --- |
| ROC-AUC | A comprehensive metric that measures a model's ability to discriminate between positive and negative classes. A value closer to 1 indicates stronger overall discriminative power. |
| PR-AUC | A comprehensive metric that measures a model's ability to identify the positive class. It is more informative than ROC-AUC when the number of negative samples greatly exceeds the positives (imbalanced data). |
| Sensitivity (Recall) | The proportion of actual positive samples that the model correctly identifies. It measures the ability to "find all the positives" (avoid missing cases). |
| PPV | The proportion of samples predicted as positive that are correct. It measures the accuracy of positive predictions. |
| NPV | The proportion of samples predicted as negative that are correct. It measures the confidence in ruling out a condition. |
| F1-Score | The harmonic mean of Sensitivity and PPV. It is a balanced score between the two, useful when both "finding positives" and "accurate predictions" are important. |
| Permutation-based Importance | Permutation-based Importance is calculated by first measuring the model's original performance (e.g., accuracy or AUC), then randomly shuffling the values of one feature to disrupt its relationship with the outcome, and finally re-evaluating the model's performance using the shuffled data. The importance of the feature is quantified by the extent to which the model's performance decreases after shuffling; a larger drop indicates a more important feature. Unlike model-specific importance metrics that rely on the model's internal structure, permutation importance is model-agnostic, directly tied to predictive performance, and generally more reliable and intuitive. |
| Shapley Additive Explanations | Based on game theory, it explains individual predictions by fairly distributing the contribution of each feature to the final outcome for that specific instance. |

**Table S3: Algorithm Overview**

| **Algorithm** | **Description** |
| --- | --- |
| Decision Tree | Decision tree is a simple and interpretable supervised learning algorithm used for classification and regression. It splits data recursively into subsets based on feature thresholds, forming a tree-like structure. While it is prone to overfitting on training data, pruning techniques can improve its performance.(Quinlan, 1986)  Hyperparameters: cost_complexity = 0.000237, tree_depth = 4, min_n = 8 |
| Logistic Regression | Logistic regression is a widely used supervised learning algorithm for binary and multiclass classification tasks. It models the probability of a target variable belonging to a specific class by applying the sigmoid function to a linear equation. Despite its simplicity, logistic regression is highly interpretable and performs well on linearly separable data.(Hosmer, Lemeshow, & Sturdivant, 2013) |
| Random Forest | Random forest is an ensemble learning algorithm based on decision trees. It builds multiple decision trees during training and combines their predictions to improve accuracy and reduce overfitting. It is robust to noise and provides insights into feature importance.(Breiman, 2001)  Hyperparameters: mtry = 6, trees = 500, min_n = 50 |
| SVM (Support Vector Machine) | SVM is a powerful supervised learning algorithm for classification and regression tasks. It finds an optimal hyperplane in a high-dimensional space to separate different classes. SVM is effective in handling small datasets and high-dimensional spaces, particularly when combined with kernel functions for nonlinear problems.(Cristianini & Shawe-Taylor, 2000)  Hyperparameters: cost = 32, rbf_sigma = 0.0001 |
| XGBoost (Extreme Gradient Boosting) | XGBoost is an efficient, flexible, and high-performance gradient boosting framework widely used for regression, classification, and ranking tasks. It minimizes prediction error by iteratively optimizing decision trees and effectively handles complex nonlinear relationships between features. With its fast computation capabilities and exceptional predictive performance, XGBoost has gained widespread adoption in both academic research and industrial applications.(Chen & Guestrin, 2016)  Hyperparameters: mtry = 6, min_n = 6, tree_depth = 3, learn_rate = 0.00891, loss_reduction = 0.692, sample_size = 0.889 |

**Table S4. Description of Participant Clinical Characteristics**

| **Vars** | **Levels** | **Overall**  **(n=820)** | **without SA**  **(n=521)** | **With SA**  **(n=299)** | **p** | **Training Set(n=706)** | **External Validation Set(n=114)** | **p** |
| --- | --- | --- | --- | --- | --- | --- | --- | --- |
| Age, mean (SD) |  | 14.67 (1.69) | 14.85 (1.71) | 14.35 (1.61) | <0.001 | 14.69 (1.70) | 14.53 (1.66) | 0.331 |
| BMI z-score, mean (SD) |  | 0.06 (1.18) | 0.09 (1.19) | 0.03 (1.17) | 0.511 | 0.11 (1.17) | -0.21 (1.20) | 0.008 |
| Depression, No. (%) | Mild | 70 (8.5) | 56 (10.7) | 14 (4.7) | <0.001 | 62 (8.8) | 8 (7.0) | 0.33 |
|  | Moderate | 234 (28.5) | 177 (34.0) | 57 (19.1) |  | 195 (27.6) | 39 (34.2) |  |
|  | Severe | 516 (62.9) | 288 (55.3) | 228 (76.3) |  | 449 (63.6) | 67 (58.8) |  |
| Anxiety, No. (%) | Normal | 72 (8.8) | 55 (10.6) | 17 (5.7) | <0.001 | 66 (9.3) | 6 (5.3) | 0.128 |
|  | Mild | 170 (20.7) | 128 (24.6) | 42 (14.0) |  | 153 (21.7) | 17 (14.9) |  |
|  | Moderate | 277 (33.8) | 185 (35.5) | 92 (30.8) |  | 234 (33.1) | 43 (37.7) |  |
|  | Severe | 301 (36.7) | 153 (29.4) | 148 (49.5) |  | 253 (35.8) | 48 (42.1) |  |
| PSQI^a^, mean (SD) |  | 11.25 (3.52) | 10.71 (3.45) | 12.18 (3.45) | <0.001 | 11.18 (3.52) | 11.63 (3.54) | 0.208 |
| Left-behind child, No. (%) | No | 336 (41.0) | 229 (44.0) | 107 (35.8) | 0.027 | 282 (39.9) | 54 (47.4) | 0.164 |
|  | Yes | 484 (59.0) | 292 (56.0) | 192 (64.2) |  | 424 (60.1) | 60 (52.6) |  |
| NSSI^b^, No. (%) | No | 267 (32.6) | 222 (42.6) | 45 (15.1) | <0.001 | 239 (33.9) | 28 (24.6) | 0.063 |
|  | Yes | 553 (67.4) | 299 (57.4) | 254 (84.9) |  | 467 (66.1) | 86 (75.4) |  |
| Disclosed suicide ideation, No. (%) | No | 339 (41.3) | 249 (47.8) | 90 (30.1) | <0.001 | 298 (42.2) | 41 (36.0) | 0.249 |
|  | Yes | 481 (58.7) | 272 (52.2) | 209 (69.9) |  | 408 (57.8) | 73 (64.0) |  |
| Grade, No. (%) | High School | 392 (47.8) | 283 (54.3) | 109 (36.5) | <0.001 | 340 (48.2) | 52 (45.6) | 0.866 |
|  | Middle School | 391 (47.7) | 218 (41.8) | 173 (57.9) |  | 334 (47.3) | 57 (50.0) |  |
|  | Other | 37 (4.5) | 20 (3.8) | 17 (5.7) |  | 32 (4.5) | 5 (4.4) |  |
| Occupation of father, No. (%) | Business | 116 (14.1) | 75 (14.4) | 41 (13.7) | 0.007 | 101 (14.3) | 15 (13.2) | 0.003 |
|  | Enterprise | 128 (15.6) | 98 (18.8) | 30 (10.0) |  | 123 (17.4) | 5 (4.4) |  |
|  | Other | 310 (37.8) | 185 (35.5) | 125 (41.8) |  | 256 (36.3) | 54 (47.4) |  |
|  | Worker | 266 (32.4) | 163 (31.3) | 103 (34.4) |  | 226 (32.0) | 40 (35.1) |  |
| Residence before age 16, No. (%) | City | 241 (29.4) | 154 (29.6) | 87 (29.1) | 0.837 | 210 (29.7) | 31 (27.2) | <0.001 |
|  | Rural | 145 (17.7) | 89 (17.1) | 56 (18.7) |  | 108 (15.3) | 37 (32.5) |  |
|  | Town | 434 (52.9) | 278 (53.4) | 156 (52.2) |  | 388 (55.0) | 46 (40.4) |  |
| Suicide attempt, No. (%) | No | NA | NA | NA | NA | 454 (64.3) | 67 (58.8) | 0.301 |
|  | Yes | NA | NA | NA |  | 252 (35.7) | 47 (41.2) |  |

^a^Pittsburgh Sleep Quality Index; ^b^Non-Suicidal Self-Injury.

**Table S5: Performance evaluation metrics of XGBoost on the external validation set.**

| **Metric** | **Mean** | **SD** | **CI_Lower** | **CI_Upper** |
| --- | --- | --- | --- | --- |
| Sensitivity | 0.85 | 0.07 | 0.71 | 1.00 |
| Specificity | 0.48 | 0.08 | 0.31 | 0.63 |
| PPV | 0.53 | 0.08 | 0.38 | 0.68 |
| NPV | 0.82 | 0.09 | 0.64 | 1.00 |
| F1 Score | 0.65 | 0.07 | 0.51 | 0.70 |

The results were validated through 1000 bootstrap resamples.


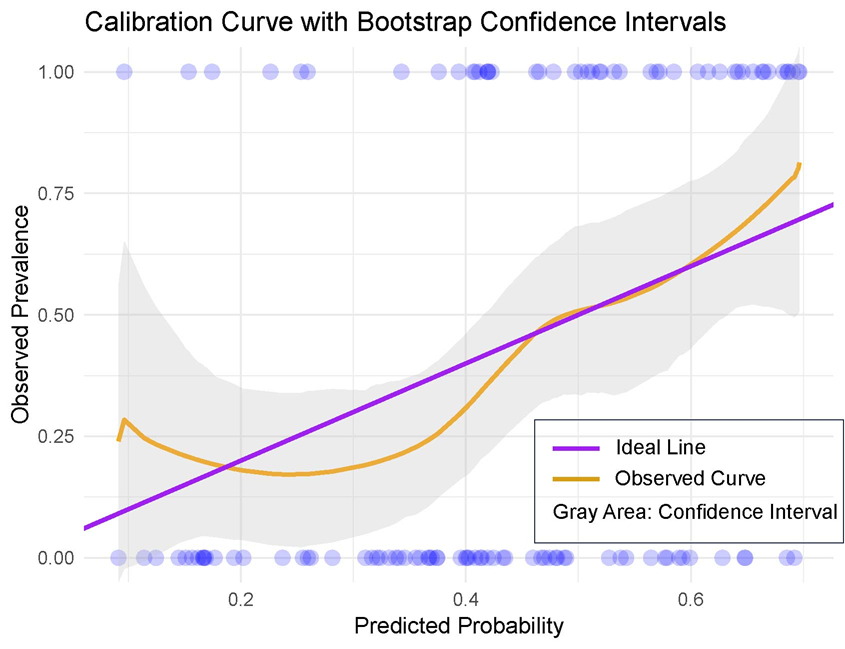


**Figure S2: Calibration curve with bootstrap confidence intervals.**

ICI (Integrated Calibration Index): 0.0576


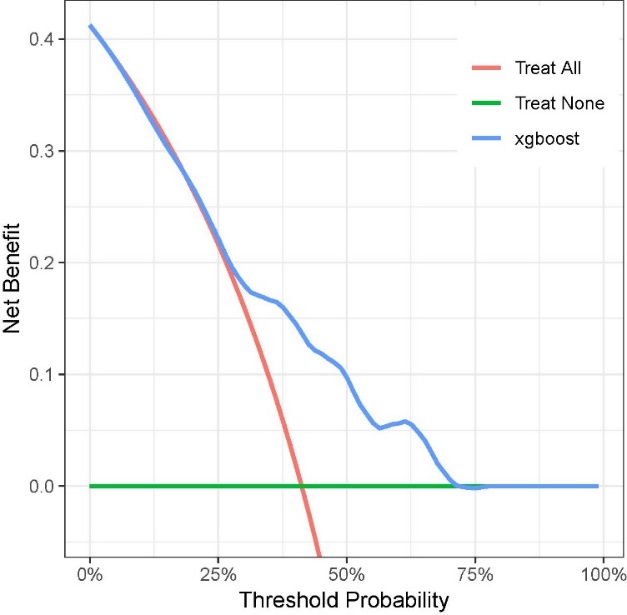
**Figure S3: Clinical decision curve analysis**

**eReferences**

Breiman, L. (2001). Random Forests. *Machine Learning*, *45*(1), 5–32.

Chen, T., & Guestrin, C. (2016). XGBoost: A scalable tree boosting system. *Proceedings of the 22nd ACM SIGKDD International Conference on Knowledge Discovery and Data Mining* (pp. 785–794). Presented at the KDD ’16: The 22nd ACM SIGKDD International Conference on Knowledge Discovery and Data Mining, San Francisco California USA: ACM. Retrieved November 22, 2024, from https://dl.acm.org/doi/10.1145/2939672.2939785

Cristianini, N., & Shawe-Taylor, J. (2000). *An introduction to support vector machines: And other kernel-based learning methods*. Cambridge: Cambridge University Press.

Hosmer, D. W., Lemeshow, S., & Sturdivant, R. X. (2013). *Applied logistic regression*. Wiley Series in Probability and Statistics (1st ed.). Wiley. Retrieved November 22, 2024, from https://onlinelibrary.wiley.com/doi/book/10.1002/9781118548387

Quinlan, J. R. (1986). Induction of decision trees. *Machine Learning*, *1*(1), 81–106.
